# Incidental finding of didelphys uterus with twin pregnancy in each cavity; A rare case report

**DOI:** 10.1016/j.ijscr.2025.110943

**Published:** 2025-01-24

**Authors:** Animut Fetene Zeleke, Mesfin Ayalew Tsegaye, Tsehaynesh Bayih Geremew, Ananya Agumasie Dessie, Tseganesh Mekonnen Hailemariam, Bethlehem Workneh Delelegn

**Affiliations:** a Dilla University, College of Medicine and Health Sciences, Department of Internal Medicine, Dilla, Ethiopia; b Dilla University, College of Medicine and Health Sciences, Department of Obstetrics and Gynecology, Dilla, Ethiopia; c Wolaita Sodo University, College of Health Sciences, Department of Surgery, Wolaita Sodo, Ethiopia; d Bahidar University, College of Health Sciences, Department of Obstetrics and Gynecology, Bahirdar, Ethiopia; e Addis Ababa University, College of Health Sciences, Department of Internal Medicine, Addis Ababa, Ethiopia

**Keywords:** Uterus didelphys, Twin pregnancy, Cesarean section, Mullerian duct anomalies

## Abstract

**Introduction and importance:**

Uterine didelphys is a Müllerian duct anomaly with two uteri and cervices, with or without a vaginal septum. A di-cavitary twin pregnancy in a uterus didelphys is an infrequent occurrence.

**Case presentation:**

A 27-year-old woman, gravida 3, para 2, at a gestational age of 37 weeks and 4 days, presented with pushing-down pain. A primary lower uterine segment cesarean section was performed due to fetal bradycardia, and incidentally, a second gravid uterus was discovered, necessitating another lower uterine segment cesarean section. Post-operative reevaluation revealed a double cervix but no vaginal septum.

**Clinical discussion:**

Uterine didelphys is a rare Müllerian duct anomaly with two uteri and cervices, often leading to complications during pregnancy. This anomaly develops from the failure of the Müllerian ducts to fuse, constituting approximately 5–11 % of all Müllerian duct anomalies. Pregnancies in women with didelphys uterus face increased risks, particularly with twin gestations, which occur at a rate of about 1 in 1,000,000. Management strategies must be individualized, considering factors such as fetal presentation and maternal preferences, with vaginal delivery being an option when possible. Early detection through appropriate imaging techniques is crucial.

**Conclusion:**

Routine follow-ups should actively search for any uterine anomalies. The management of such cases requires individualized care tailored to each specific situation.

## Highlights

• Di-cavitary twin pregnancy in uterine didelphys is extremely rare.
• Uterine didelphys is not a direct indication for a cesarean section.
• Di-cavitary twin pregnancy in the didelphys uterus requires an individualized plan.

## 1 Introduction

Müllerian duct anomalies (MDAs) are congenital defects resulting from abnormal embryological development of the Müllerian ducts during the 6th to 22nd week of fetal life. These developmental abnormalities can include failure of development, fusion, canalization, or reabsorption, leading to various forms of MDAs such as the septate uterus, bicornuate uterus, arcuate uterus, unicornuate uterus, didelphys uterus, and complete agenesis. The overall incidence of MDAs is estimated to be up to 7 % in the general population, and up to 25 % in those with a history of both infertility and miscarriage. [[1], [2], [3], [4], [5]]

Uterus didelphys is characterized by the presence of two uteri and two cervices. In addition there may or may not be longitudinal vaginal septum. [6] This arises from a lack of fusion of the Müllerian ducts. [7] In some instances, it may coexist with obstructed hemivagina and renal agenesis, also known as Herlyn-Werner-Wunderlich (HWW) syndrome. [1,7,8] Uterus didelphys is a rare form of MDA, accounting for 5–11 % of all MDAs. [3,7,8]

Pregnancies occurring in uteri with MDAs are associated with an increased risk of complications [2,8,9]. This risk is further amplified in cases of twin pregnancies within both cavities, which is exceedingly rare, occurring at a rate of approximately 1 in 1,000,000 pregnancies. There are only a few documented case reports detailing such occurrences [6,10].

This case report presents a mother with a history of two spontaneous abortions who was found to have a twin pregnancy—one fetus in each uterine cavity—discovered incidentally during a cesarean section performed due to fetal bradycardia.

This case report was prepared following the SCARE guidelines. [11]

Case presentation.

This case report describes a 27-year-old woman, Gravida 3, Abortion 2 (one in the first trimester and one in the second trimester), who presented at a gestational age of 37 weeks and 4 days with pushing-down pain lasting for 5 h at the labor and delivery unit. The intensity and frequency of her pain gradually increased, lasting approximately 30–35 s and occurring every 3–5 min. Although she had regular antenatal care follow-ups, uterine didelphys was not diagnosed due to a limited ultrasound evaluation.

Upon physical examination, the patient had a gravid uterus consistent with 38 weeks of gestation, and fetal heartbeat was detected at a rate of 142 bpm. She experienced one contraction in 10 min lasting about 20 s. The cervix was admitting one finger with 50 % effacement. After four hours of evaluation, cervical dilation progressed to 5 cm with 80 % effacement, although the fetal station remained high. Due to persistent fetal bradycardia, with heart rates between 70 and 98 bpm, a cesarean section was performed. A male neonate weighing 2000 g was delivered with APGAR scores of 8 and 10 at the first and fifth minutes, respectively.

**Fig. 1:**
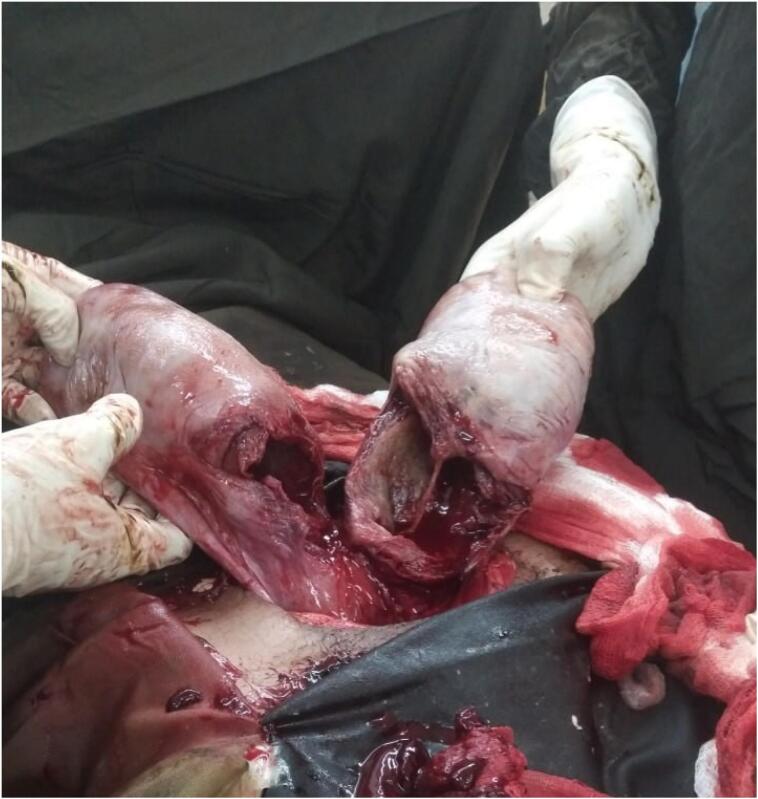
A picture of uterus didelphys, both uteri exteriorized with their lower segment transverse incision

During the procedure, a second gravid uterus was identified incidentally, leading to a second cesarean section (Fig. 1) where a female neonate weighing 2400 g was delivered with APGAR scores of 7 and 10 at the first and fifth minutes, respectively. Both uteri were closed using continuous sutures in double layers, and there was no excessive bleeding or atony during the operation. Following the suturing, uterine didelphys was considered. A vaginal and cervical reevaluation revealed a single vagina without any septum but confirmed the presence of two cervices. An abdominal ultrasound was conducted to check for renal agenesis, which returned normal findings with both kidneys in normal size and shape. The patient was scheduled for a postpartum visit and discharged in good condition.

The patient had menarche at age 15, with regular menstrual cycles. She experienced dysmenorrhea before marriage but reported improvement after initiating sexual activity. Additionally, she had two consecutive spontaneous abortions during early pregnancy.

## 2 Discussion

Uterine didelphys is typically asymptomatic, but when symptoms do occur, they may include dysmenorrhea, dyspareunia, and, in rare cases, hematocolpos or hematometracolpos. [1,4,7,10] In this patient, a history of dysmenorrhea was noted; however, she did not report any episodes of dyspareunia.

The diagnosis of a didelphys uterus and other Müllerian duct anomalies (MDAs) relies on both clinical evaluation and imaging findings, often supplemented by additional procedures. Abdominopelvic ultrasound is usually the first imaging modality. Magnetic resonance imaging (MRI) is a key imaging modality because it can delineate pelvic structures and identify concomitant anomalies. Other diagnostic options include hysteroscopy, hysterosalpingography (HSG), and laparoscopy. [1,3,4,6]

Fertility outcomes in women with a didelphys uterus are generally better than those with other types of MDAs but still inferior to those with a normal uterus [1,8]. Some studies have indicated that outcomes may be comparable to those of other MDAs [1]. Women with MDAs face an increased risk of spontaneous abortion, which may explain this patient's history of two previous spontaneous abortions [1,2,11].

Pregnancies occurring in utero with MDAs are complicated with miscarriage, premature birth, malpresentation, fetal growth restriction, ectopic pregnancy, and a heightened risk of cesarean section (CS). The increased CS rate is primarily attributed to malpresentation and labor dystocia rather than being a direct indication for surgical delivery. [1,2,6,12,13]

Twin pregnancies in a uterus didelphys—where each fetus occupies a separate cavity—are exceedingly rare, occurring at an estimated rate of 1 in 1,000,000 pregnancies. This scenario presents unique challenges for obstetricians due to the limited number of documented cases. Delayed interval delivery has been reported in such cases due to the independent functioning of each uterus and cervix [ 10,12].

While a didelphys uterus does not inherently necessitate cesarean delivery, vaginal delivery should be considered first based on several factors: the number of gestations, fetal presentation, previous delivery methods, and maternal preferences. However, the presence of a thick and inelastic vaginal septum may warrant a cesarean section due to the increased risk of dystocia. [1,4,6,10,12] Trials of labor after cesarean (TOLAC) for singleton pregnancies have shown success in both scarred and unscarred uteri. In conclusion, managing twin pregnancies within separate cavities of a didelphys uterus requires careful consideration due to the distinct anatomical challenges presented by this condition. [12]

## 3 Conclusion

Didelphys uterus is a rare uterine anomaly, and dicavitary twin pregnancy in a didelphys uterus is extremely rare. Searching for any uterine structural anomaly in the ANC contact is useful. Delivery planning in uterus didelphys must be individualized, taking into account fetal presentation and maternal preferences, with consideration given to vaginal delivery when appropriate.

All authors have approved the final submitted manuscript.

## Author contribution

Animut Fetene Zeleke, MD; contributed to patient care, the conception of the case report, acquiring and interpreting the data, drafting the manuscript, undertaking the literature review, and revising the article critically for important intellectual content.

Mesfin Ayalew Tsegaye, MD; contributed to patient care, the conception of the case report, acquiring and interpreting the data, drafting the manuscript, undertaking the literature review, and revising the article critically for important intellectual content.

Tsehaynesh Bayih Geremew(IESO); contributed to patient care, the conception of the case report, drafting the manuscript, and revising the article critically for important intellectual content.

Ananya Agumasie Dessie(MD); contributed to patient care, acquiring and interpreting the data, undertaking the literature review, and revising the article critically for important intellectual content.

Tseganesh Mekonnen Hailemariam, MD; contributed to the conception of the case report, acquiring and interpreting the data, drafting the manuscript, and revising the article critically for important intellectual content.

Bethlehem Workneh Delelegn, MD; the conception of the case report, acquiring and interpreting the data, and drafting the manuscript.

## Patient consent

Written informed consent was obtained from the patient for publication of this case report and accompanying images.

## Ethical approval

Ethical approval is exempted for case reports by Dilla University Hospital.

## Guarantor

Mesfin Ayalew Tsegaye.

## Provenance and peer review

This article was not commissioned and was peer-reviewed.

## Funding

This work did not receive any specific grant from funding agencies in the public, commercial, or not-for-profit sectors.

## Declaration of competing interest

The authors declare that they have no conflict of interest regarding the publication of this case report.
